# Using electronic patient records to assess the effect of a complex antenatal intervention in a cluster randomised controlled trial—data management experience from the DESiGN Trial team

**DOI:** 10.1186/s13063-021-05141-8

**Published:** 2021-03-08

**Authors:** Sophie Relph, Maria Elstad, Bolaji Coker, Matias C. Vieira, Natalie Moitt, Walter Muruet Gutierrez, Asma Khalil, Jane Sandall, Andrew Copas, Deborah A. Lawlor, Dharmintra Pasupathy, on behalf of the DESIGN Trial team, Kirstie Coxon, Andrew Healey, Alessandro Alagna, Annette Briley, Mark Johnson, Christoph Lees, Neil Marlow, Lesley McCowan, Louise Page, Donald Peebles, Andrew Shennan, Basky Thilaganathan

**Affiliations:** 1 Department of Women and Children’s Health, School of Life Course Sciences, Faculty of Life Sciences and Medicine, King’s College London, 10th Floor North Wing, St. Thomas’ Hospital, Westminster Bridge Road, London, SE1 7EH UK; 2 School of Population Health and Environmental Sciences, Faculty of Life Sciences and Medicine, King’s College London, 4th Floor, Addison House, Guy’s Campus, London, SE1 1UL UK; 3 Division of Health and Social Care Research, King’s College London, London, UK; 4 NIHR Biomedical Research Centre at Guy’s and St Thomas’ NHS Foundation Trust and King’s College London, Guy’s Hospital, London, UK; 5 Department of Obstetrics and Gynaecology, University of Campinas (UNICAMP), School of Medical Sciences, 101 Alexander Fleming St, Cidade Universitaria, Campinas, SP Brazil; 6 Fetal Medicine Unit, St George’s University Hospitals NHS Foundation Trust, Blackshaw Road, London, SW17 0QT UK; 7 Molecular & Clinical Sciences Research Institute, St George’s, University of London, Cranmer Terrace, London, SW17 0RE UK; 8 Centre for Pragmatic Global Health Trials, Institute for Global Health, University College London, Gower Street, London, WC1E 6BT UK; 9 Population Health Science, Bristol Medical School, University of Bristol, Bristol, BS8 2BL UK; 10 Bristol NIHR Biomedical Research Centre, Bristol, BS8 2BL UK; 11 MRC Integrative Epidemiology Unit at the University of Bristol, Bristol, BS8 2BL UK; 12 Speciality of Obstetrics, Gynaecology and Neonatology, Westmead Clinical School, Faculty of Medicine and Health, University of Sydney, Sydney, Australia

**Keywords:** Data management, Data linkage, Methodology, Electronic patient records, Cluster randomised trial, Perinatal, Maternal

## Abstract

**Background:**

The use of electronic patient records for assessing outcomes in clinical trials is a methodological strategy intended to drive faster and more cost-efficient acquisition of results. The aim of this manuscript was to outline the data collection and management considerations of a maternity and perinatal clinical trial using data from electronic patient records, exemplifying the DESiGN Trial as a case study.

**Methods:**

The DESiGN Trial is a cluster randomised control trial assessing the effect of a complex intervention versus standard care for identifying small for gestational age foetuses. Data on maternal/perinatal characteristics and outcomes including infants admitted to neonatal care, parameters from foetal ultrasound and details of hospital activity for health-economic evaluation were collected at two time points from four types of electronic patient records held in 22 different electronic record systems at the 13 research clusters. Data were pseudonymised on site using a bespoke Microsoft Excel macro and securely transferred to the central data store. Data quality checks were undertaken. Rules for data harmonisation of the raw data were developed and a data dictionary produced, along with rules and assumptions for data linkage of the datasets. The dictionary included descriptions of the rationale and assumptions for data harmonisation and quality checks.

**Results:**

Data were collected on 182,052 babies from 178,350 pregnancies in 165,397 unique women. Data availability and completeness varied across research sites; each of eight variables which were key to calculation of the primary outcome were completely missing in median 3 (range 1–4) clusters at the time of the first data download. This improved by the second data download following clarification of instructions to the research sites (each of the eight key variables were completely missing in median 1 (range 0–1) cluster at the second time point). Common data management challenges were harmonising a single variable from multiple sources and categorising free-text data, solutions were developed for this trial.

**Conclusions:**

Conduct of clinical trials which use electronic patient records for the assessment of outcomes can be time and cost-effective but still requires appropriate time and resources to maximise data quality. A difficulty for pregnancy and perinatal research in the UK is the wide variety of different systems used to collect patient data across maternity units. In this manuscript, we describe how we managed this and provide a detailed data dictionary covering the harmonisation of variable names and values that will be helpful for other researchers working with these data.

**Trial registration:**

Primary registry and trial identifying number: ISRCTN 67698474. Registered on 02/11/16.

**Supplementary Information:**

The online version contains supplementary material available at 10.1186/s13063-021-05141-8.

## Background

Electronic patient record (EPR) systems, also known as electronic medical records, store data that are routinely recorded during the course of normal clinical investigation and management, for the purpose of documenting the events of the patient visit. In the UK, EPR systems store relevant data on patients’ demographics, medical and surgical history and records of consultations which have occurred with any healthcare provider at the site at which the record is stored. EPR systems may also store appointment or admission data, medication history or a record of investigations, although different specialist systems are often used for different types of data or clinical tasks (e.g. prescribing, or reporting on radiographic investigations).

The use of EPR data for analysis in clinical trials is a strategy intended to support more cost- and time-efficient trials [1]. Whilst there are many examples of national epidemiological research and audit conducted using EPR data over the past 20 years [2], clinical trials using data collected from EPR have only become more prevalent in the last 5 years. Examples in maternal and perinatal medicine come from Canada [3], Sweden [4], the Netherlands [5], Scotland [6, 7], Wales [8] and England [9, 10].

The DESiGN (DEtection of the Small for GestatioNal age fetus) Trial is a cluster randomised control trial which used data primarily from EPR to test the clinical impact and cost-effectiveness of a complex antenatal intervention [10]. This approach was chosen as a cost- and time-effective, feasible method to collect data on baseline characteristics, and primary and secondary clinical and economic outcomes of approximately 180,000 births at 13 cluster sites over the 3-year data collection period. The trial was closed following completed follow-up of outcomes in February 2019.

The aim of this manuscript was to outline the data collection and management considerations of a maternity and perinatal clinical trial using EPR data, exemplifying the DESiGN Trial as a case study that others could learn from.

## Methods

### Study description

The aim of the DESiGN trial is to test the clinical effectiveness and cost efficacy of the Growth Assessment Protocol (GAP) [11], compared with standard practice, to accurately detect small for gestational age (SGA) foetuses. It will also assess secondary clinical outcomes, cost-effectiveness and, as part of a process evaluation, the implementation of the GAP.

Cluster randomisation was chosen because the intervention required implementation at the maternity unit level. It required staff training and implementation of protocols across the site. The designated clusters were maternity units. Pregnant women being cared for in each maternity unit were expected to be exposed to the intervention or standard treatment, as per allocation arm.

The primary outcome was antenatal ultrasound detection of SGA in babies found to be SGA at birth. Secondary outcomes included maternal labour and birth outcomes, neonatal outcomes, implementation outcomes and an economic evaluation of the intervention cost-effectiveness. The full protocol of the study has been published previously [10].

From the outset of this trial, data collection was planned to use downloaded reports from established EPR systems holding maternal, foetal and/or neonatal data, including foetal measurements taken at ultrasound and hospital administrative data on inpatient admissions and outpatient appointments. Data were required from three trial phases: for 1 year pre-randomisation (to study baseline characteristics and use these to adjust outcomes across clusters), implementation washout (a variable time period during which the sites allocated to the intervention were preparing for and implementing the intervention), trial comparison phase (a 4–6-month period during which all births in a site contributed to the primary analysis of the trial outcomes and the care continued as allocated).

**Fig. 1:**
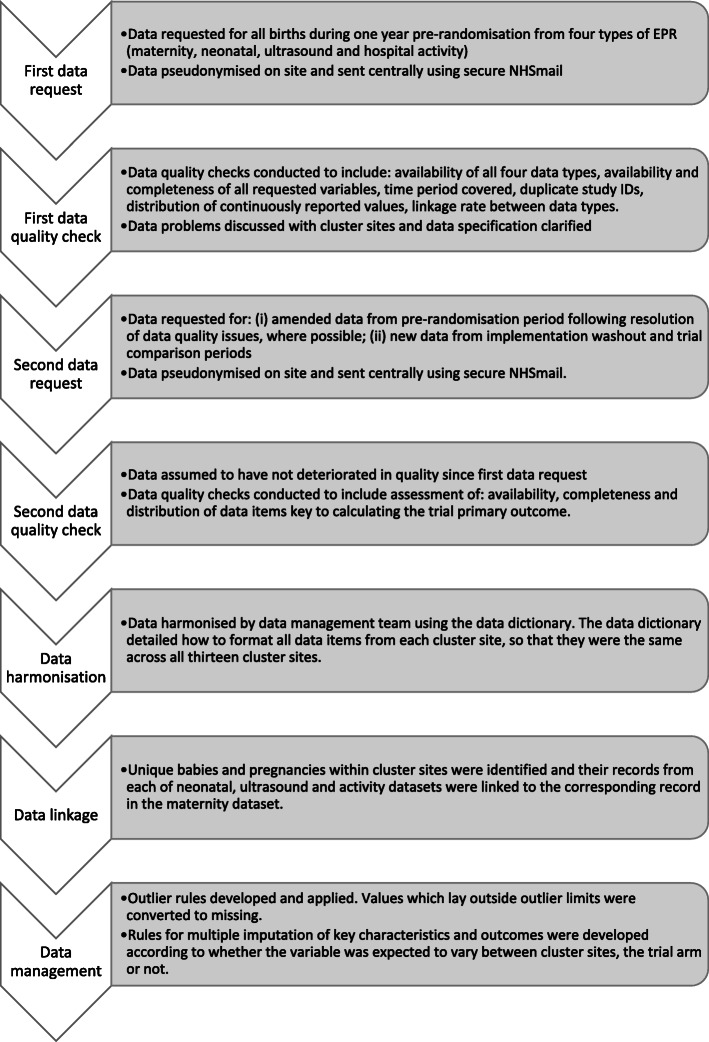
Flowchart of the summarised data management process

All data management for this trial was conducted using StataIC v15 (StataCorp LP, College Station, Texas). The full data management process has been summarised in Fig. 1.

### Ethical review

Ethical review of the protocol was conducted by the Bloomsbury Research Ethics Council (Ref: 12/LO/1632) and UK Confidentiality Advisory Group (CAG Ref: 12/CAG/0195). Ethical review by CAG was required for the use of women’s EPR data without direct consent (opt-out basis) and so that the research team were able to access patient-identifiable data at the clinical research sites. This was essential for accurate linkage of data from different EPR systems, through pseudonymisation processes.

### Data acquisition

**Fig. 2:**
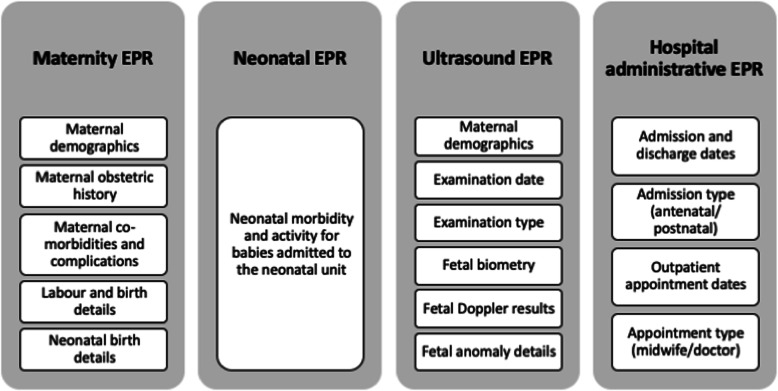
Expected data types and sources

**Fig. 3:**
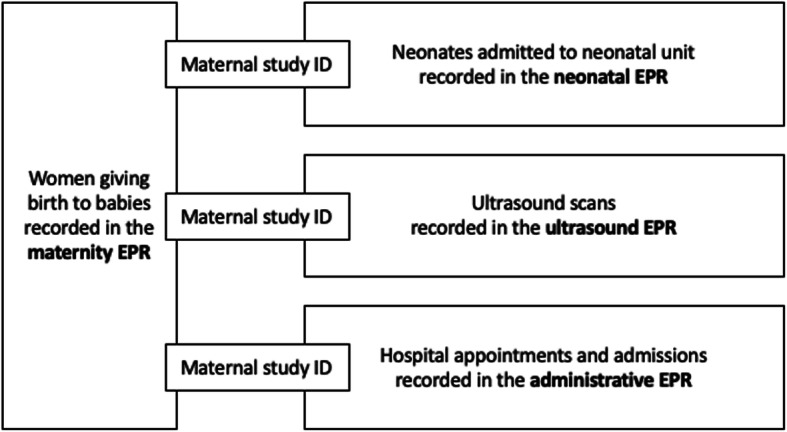
Planned linkage of the four datasets

Data were required from four types of EPR system: (i) data on maternal/perinatal characteristics and outcomes (maternity EPR), (ii) data on characteristics and outcomes of infants admitted to neonatal care (neonatal EPR), (iii) data on timing and findings of antenatal ultrasound scans (ultrasound EPR) and (iv) data on hospital activity, e.g. number of antenatal clinic visits (hospital administrative EPR). These data were collected at two time points from a key point of contact (usually a research midwife) at the 13 cluster sites. Data were requested in Microsoft Excel spreadsheets. The first request was initiated in January 2018 for data on births during the 1 year prior to randomisation of sites into the trial, to allow for data quality checks and troubleshooting prior to the subsequent request. The second request was initiated in March 2019. This request was for a re-run of any data download for the pre-randomisation phase that had been amended following resolution of quality issues from the first request and for data from the implementation washout and trial comparison phases. The types of data and expected source EPR system at each site are summarised in Fig. 2. The maternity EPR acted as a spine onto which the other three data types were to be linked (Fig. 3).

Since the neonatal data were expected to come from the same EPR software (CleverMed Badgernet Neonatal) in all units, a data extraction tool was built, specific to that software (the code is available in Additional File 1). This was intended to assist the site clinicians in quickly extracting the relevant neonatal data for the study. It was also possible to share pre-built data extraction tools for two of the ultrasound EPR software types (Viewpoint, GE Healthcare and Astraia).

### Pseudonymisation

Data provided by the maternity sites included patient identifiers: National Health Service (NHS) patient number, hospital patient number, date of birth (DOB) and postcode. Patient identifiers were required so that a unique study identifier (ID) could be generated for each woman in the study across all linked datasets, using a pseudonymisation tool.

The pseudonymisation tool was developed by the research team as an Excel macro using Microsoft Visual Basic for Applications and refined and tested using simulated data in Stata v15 to prevent generation of duplicate study identifiers. The simulation dataset included fictitious NHS numbers and DOB for women, and dates of delivery (DOD) for the infants. All simulated women had DOB between 1 January 1989 and 31 December 1990 and DOD between 1 January 2017 and 30 November 2018. Narrow date ranges were chosen to increase the number of duplicate dates and therefore test the risk that different women could be allocated the same study ID.

On site, the pseudonymisation procedure was conducted by the trial clinical fellow and data manager, under the supervision of the cluster’s key clinical contact, in keeping with ethical approval for data flow. A manually produced pseudonym was generated if a woman did not have any of the variables needed by the pseudonymisation tool.

The algorithm for the pseudonymisation tool cannot be shared because of the risk of de-anonymisation.

### Data extraction and storage

Following generation of a pseudonymised ID, the women’s DOB were also used to calculate their age at delivery of the neonate and their postcodes were used to generate measures of socioeconomic deprivation (index of multiple deprivation, lower layer and middle layer super output areas) using the National Statistics Postcode look-up tables [12]. Following this, all identifiable data (NHS/hospital numbers, maternal DOB and postcodes) were removed from the pseudonymised dataset.

The pseudonymised ID allowed early linkage between the four datasets. Women and babies who featured in the neonatal, ultrasound or activity datasets, but did not have a record of a birth in the maternity dataset were identified and removed from these three datasets. Later linkage was conducted to link unique pregnancies across the four datasets, for women who had more than one pregnancy during the study period.

All pseudonymised datasets were checked for absence of patient identifiers by the trial research fellow (SR), trial data manager (ME) and local clinical contact before the data were electronically transferred back to the central research site using NHS Digital’s secured email system, NHSmail [13], and stored on the servers based at King’s College London. The keys linking the newly generated study ID with the women’s identifiers were left with the clinical contact, to be stored for a minimum of 5 years.

### Initial assessment of data quality and completeness

The data collected at the first download (for the pre-randomisation trial phase) underwent the following checks for completeness and plausibility:

- Presence of all four requested datasets (maternity, neonatal, ultrasound and activity data).
- Duplication of study IDs includes assessment of whether these were true duplicates, or the same woman with multiple birth(s).
- Matching of study IDs across the 4 datasets, i.e. how many of the study IDs from the maternal data appeared in the linked datasets.
- Presence of the requested variables in the dataset
- Level of completeness of the requested variables
- Range, median, 5th, 25th, 75th and 95th centile of the continuously reported variables, e.g. maternal age, height, weight.
- Date range of all reported births and hospital activities—checking these were within the requested timeframe.

Where data quality issues could be rectified, these were addressed with the cluster site before the second data download. If data quality issues could not be resolved, these were recorded.

The second data download was then subject to a limited data quality checklist (it was assumed that the data quality had not deteriorated since the first download):

- Resolution of any data quality issues raised from data quality checks on the data downloaded following the first request
- Assessment for duplicate study IDs
- Completeness of the variables which are key to the calculation of the trial primary outcome.
- Distribution of the continuous variables required to calculate the primary outcome (as per assessment of numerical distribution above)
- Value responses to categorical variables required to calculate the primary outcome
- Date range of all reported births and hospital activity data

### Data harmonisation

In order to harmonise the four datasets from the different EPR systems, a data dictionary was developed using the list of requested variables. This was intended to guide the data management team in the harmonisation process by listing the abbreviated and full variable names, a description of the variable and, for each research cluster, a guide to the re-categorisation of textual data (e.g. mode of birth) or re-calculation of units for numerical data. The full data dictionary can be viewed in Additional File 2.

**Fig. 4:**
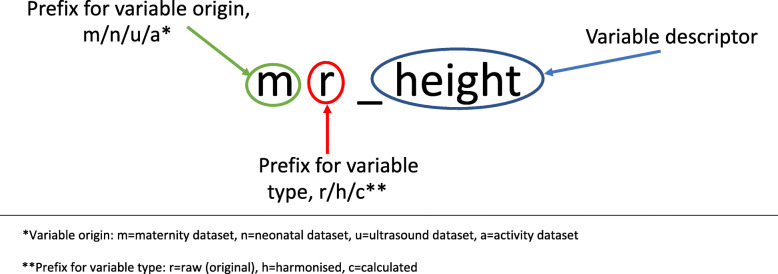
Standardised nomenclature for data dictionary

A standardised nomenclature (Fig. 4) was used for the abbreviated variable names to identify the source of the data (e.g. maternal or neonatal EPR systems) and the degree of data management which had been done to the variable (e.g. raw, harmonised or calculated variable).

For continuous numerical variables, guidance was provided on the appropriate units to use, and how to calculate where necessary (e.g. if height was provided in feet and inches, it was converted to metres and then used to calculate body mass index, BMI).

For categorical variables, guidance was produced for each site on how to change the variable to the harmonised version (e.g. how to merge the multiple descriptions of mode of birth into three categories: unassisted vaginal birth, assisted vaginal birth and caesarean section). The way in which text responses to categorical (or free text) variables were to be (re)categorised was decided in advance by consensus of the clinicians in the research team, following both familiarisation of the early datasets and consideration of what categories would be useful for the final planned analyses. Where possible, variables were re-categorised as binary, e.g. pre-existing hypertension: ‘yes’/‘no’. In all cases, but particularly important where it was not possible to re-categorise a variable according to the pre-planned categories, the raw data were also kept in the final dataset. The main clinical research team (SR, MCV, DP) familiarised themselves with the available data formats and agreed rules for data harmonisation through consensus, in advance of the active data management processes.

- Within any cluster’s dataset, if only an affirmative value is recorded for a binary variable (e.g. presence or absence of chronic hypertension), the missing values were treated as negatives (i.e. no hypertension) and changed to values which reflected this. We assumed that only the affirmative option was available to the person who entered the data.
  - Where negative values were recorded as well as affirmative, missing values were left missing during data management. For some instances however, the proportion of affirmative values in the cluster was close to the proportion expected following comparison with national audit results from the same period [14] (e.g. for severe perineal trauma) suggesting missing values would almost always be negative. These missing values were not imputed and were subsequently treated as negative in analysis.
- Within any dataset, where data for clinical diagnoses or events were available from more than one raw source, any record of the value was regarded as it being positive, even if it was not recorded as positive elsewhere. For example, where a woman was recorded as having an epidural in the ‘labour anaesthesia’ variable but this was not recorded in the ‘birth anaesthesia’ variable (both from the maternity dataset), she was regarded as having had an epidural.

Harmonisation was an iterative process. Initially, only data derived from maternity EPR were harmonised. The harmonised maternity dataset (one per hospital) were then checked for adherence to the data dictionary and data management rules by a member of the clinical study team. Where errors were made, the clinician conducting the harmonisation checks sent a written list of required edits to the data management team, who effected the changes. This meant that a clear audit trail was in place for changes to the data. The same process was then repeated for the updated versions of the maternity datasets and for the ultrasound, neonatal and activity datasets, until the clinician was satisfied with the final datasets.

### Data linkage for the linkage of unique pregnancies

Data linkage to match data for the same pregnancy or infant from the neonatal, ultrasound and activity datasets with the correct woman and birth in the maternal dataset (some women had more than one pregnancy or baby) was conducted using the pseudonyms generated at data collection and the following rules, by adding a ‘_n’ suffix to the study ID (where ‘n’ refers to the *n*th pregnancy at a particular site during the whole trial period for the woman with that study ID):

- The neonates in the neonatal EPR were matched to the correct mother and pregnancy in the maternal EPR system using (i) the maternal study ID (present in both datasets) and (ii) the neonatal date of birth within 7 days of the mother’s date of delivery.
- The hospital activity within the administrative dataset was matched to the appropriate mother and pregnancy using (i) the maternal study ID (present in both datasets) and (ii) the timing of the appointment or admission: this was required to fall between the estimated date of conception (EDC = date of delivery − gestational age at birth + 14 days) and the date of delivery. For this trial, we were not collecting data on postnatal readmissions.
- The ultrasound scans within the ultrasound dataset were matched to the correct mother and pregnancy using (i) the maternal study ID (present in both datasets) and (ii) the timing of the ultrasound scan: this was required to fall between the EDC and the date of delivery.

### Assessing data quality in the linked dataset

Quantitative variables were assessed according to an outlier policy. We calculated the 3rd, 4th and 5th standard deviation limits and the 1st and 99th centiles in the data distribution for each continuous variable. These were used to highlight possible implausible values that were likely erroneous data entries. However, these distributional cut-points are arbitrary and are also influenced by whether the variable has an (approximate) normal distribution. Simply removing variables beyond these limits could result in accurate data being removed. These limits were therefore used as indicators of potential erroneous values and the final outlier limits were derived following clinician consensus on values which were sensible (Table 1). Values outside the outlier limits were converted to missing.

**Table 1:** Outlier limits derived following clinical consensus

	Lower limit	Upper limit
**Maternal age at delivery (years)**	13y	60y
**Height (cm)**	120 cm	200 cm
**Weight (kg)**	30 kg	200 kg
**BMI (kg/m**^**2**^**)**	13 kg/m^2^	70 kg/m^2^
**EBL (mL)**	1 mL	15,000 mL
**Birthweight (g)**	100 g	6000 g

Summary statistics, including variable completeness for key sociodemographic, clinical and outcome variables were produced. Data quality was compared across sites and timepoints.

### Managing missing data

Missing values were multiply imputed through chained equations (MICE) with 10 imputations under the missing-at-random assumption [15]. A common set of predictors was chosen to predict missing values in each variable, each chosen because it was expected to be a good predictor of most if not all the variables and including the primary outcome and trial phase (pre-randomisation, washout and comparison).

During the imputation process, the primary outcome was captured by a three-category variable: baby born SGA and detected by antenatal ultrasound, baby born SGA and not detected by ultrasound, and baby not born SGA. This three-category variable was imputed like any other during the imputation process, but the primary outcome finally used for analysis was calculated from its imputed components (e.g. maternal weight, neonatal birthweight, ethnicity). This approach was taken because we needed to use the primary outcome as a predictor of other variables in the imputation process but it was not feasible to repeatedly and instantly passively impute the primary outcome because identification of a baby as SGA by customised centiles at birth, and by antenatal ultrasound, requires calculations of centiles that could only be done manually using a ‘macro’ (no direct formula available).

Variables were imputed within cluster wherever possible, as characteristics of women and clinical processes were expected to vary between clusters. Parity, maternal height and maternal weight were imputed across clusters, because some clusters had high levels of missing data (and for each factor one site had no data) and the rates were not expected to vary widely between clusters. For one site, parity was only available as a binary variable (nulliparous or multiparous) but an ordinal variable was required. Parity was imputed using the common set of predictors and, at this site, predicted parity values of zero for women known to be multiparous were replaced with a pre-specified value of ‘one’.

Ethnicity had high rates of missing data at some sites but was imputed within cluster nevertheless because this factor was known to vary strongly across sites. The full list of variables imputed, whether imputed within or across clusters, and the predictors used for their imputation are summarised in Additional File 3.

Summary statistics from the imputed dataset were compared to the equivalent summary statistics from the observed data.

All assumptions and processes used were discussed and approved by the joint Trial Steering and Data Monitoring Committee.

## Results

The final data resource comprised data on 182,052 babies from 178,350 pregnancies in 165,397 unique women. It was not possible to obtain one data type (hospital administrative data) at one site at either time point; hence, the final data resource was derived from 102 datasets out of a potential 104 datasets (four datasets across two time periods at 13 research sites). Two other datasets (ultrasound data from one site in both time periods) had to be augmented with manually collected data, since the local radiology software did not support electronic reporting at scale, during the pre-randomisation and implementation washout trial phases.

The data request for the first download was submitted in February 2018 and the last research site supplied data for this download request in February 2019. The second download request was initiated in April 2019 and the last dataset was collected 6 months later, in October 2019.

### Pseudonymisation simulation

A simulation of 100,000 records using different maternal identifiers and random numbers produced a non-duplicated study ID with the fourth iteration (Table 2). Maternal NHS number and DOB were chosen as the identifiable variables due to low missingness and high reliability. The final tool (test round 4) created a 20-character study identification string from two maternal identifiable variables (NHS number and DOB) where the same pseudonym was always generated for the same patient.

**Table 2:** Refinement of the pseudonymisation tool

Test round	Maternal data components used	Study ID format	Study ID length	Duplicates
1st	DOB, DOD, NHS number	########## e.g.1234567890	10	18,382/100000
2nd	DOB, DOD, NHS number	############ e.g. 123456789012	12	18,382/100000
3rd	DOB, DOD, NHS number, check digit	###############XXX e.g. 123456789012345ABC	18	21/100000
4th	DOB, NHS number, check digit, random component with seed	###################X e.g. 1234567891234567891A	20	0/100000

*DOB* date of birth, *DOD* date of delivery (of neonate), *ID* identifier, *NHS* National Health service

Of the 165,397 unique women, 705 did not have key information required to generate an automated study pseudonym and so were given a manually produced ID. It is important to note that this manually produced ID denotes a unique pregnancy; the same woman would not be given the same ID if she had more than one birth in the whole trial period.

### Data quality—initial assessment

The data from maternity and neonatal EPR systems were provided in wide format (one row per baby born). The data from ultrasound and hospital administrative EPR systems were always provided in long format (one row per scan, appointment or hospital admission).

Common problems with the datasets obtained following the first request were:

1. Missing variables which were key to the reporting of the trial primary outcome or secondary outcomes. This triggered a request for an amended data download (at 6 of total 13 sites).
2. Duplicated records, which had to be distinguished from records for multiple births. The ultrasound data were used to clarify whether the pregnancy was a singleton pregnancy (5 of 13 sites).
3. Data reports produced on a per-pregnancy rather than per-baby basis, i.e. for women reported to have had a multiple birth, one record would be expected per baby born but often only the record for the first-born baby was provided. This triggered a request for an amended report (2 sites).

Where problems persisted with the second request for data, it was usually because particular variables were not routinely collected in the EPR.

The results of assessment for variable availability, median and range of completeness for some of the variables which were key to calculating the primary outcome of this trial (antenatal diagnosis of small growth status at birth by customised and population-derived birthweight centiles) are displayed in Table 3. The number of variables which were completely missing improved following modified or clarified data requests for the second download. The median completeness where the variable was available did not change between data requests, but the range widened in the second request, caused by availability of more variables, but with lower levels of completeness.

**Table 3:** Quality checks for subsequent requests for data on births during the pre-randomisation phase

	Request period	Maternal weight	Maternal height	Maternal ethnicity	Maternal parity	Neonatal sex	Gestational age at birth	Neonatal birthweight
**Number sites where variable was missing completely**	**First request for pre-randomisation data**	4	4	3	2	4	1	3
	**Second request for pre-randomisation**	1	1	0	1	0	0	0
**% complete at sites where variable provided (median; range)**	**First request for pre-randomisation data**	98.6% (85.6–100.0%)	98.6% (86.2–100.0%)	100.0% (67.2–100.0%)	99.2% (1.0–100.0%)	100.0% (99.2–100.0%)	100.0% (80.4–100.0%)	100.0% (99.0–100.0%)
	**Second request for pre-randomisation**	96.5% (0.6–99.8%)	96.5% (0.6–99.8%)	98.9% (23.9–100.0%)	98.1% (20.3–100.0%)	100.0% (99.1–100.0%)	99.9% (89.0–100.0%)	99.9% (99.1–100.0%)

### Data harmonisation

The 22 EPR systems from which the raw data were sourced are listed in Table 4. The neonatal data were the only data type with a single EPR system source, this was reflected in the consistent formats of the data provided.

**Table 4:** EPR systems used at sites for each data type

Maternity EPR system	Ultrasound EPR system	Neonatal EPR system	Hospital administrative EPR system (appointments / admissions)
Medway Maternity K2 E3 Cerner Euroking CMIS EPR Badgernet Maternity (CleverMed)	Astraia Viewpoint (GE Healthcare) CRIS Solitorn RIS / PACS	Badgernet Neonatal (CleverMed)	Medway PAS CMIS CareCast EPR APAS OASIS iClip

### Data linkage

**Fig. 5:**
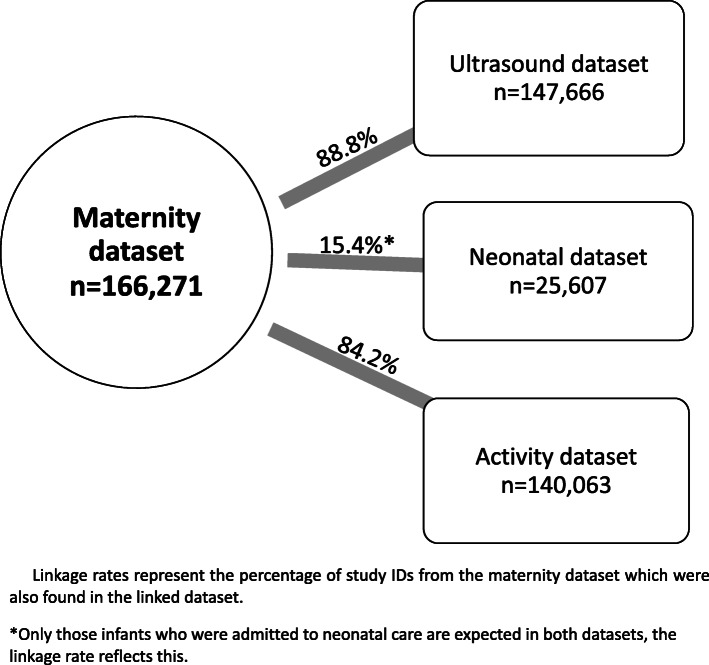
Linkage rates between the maternity and linked datasets

One hundred percent of study IDs from the linked (neonatal, ultrasound and activity) datasets matched with study IDs from the maternal dataset because the IDs in the linked datasets were derived from the maternal dataset. The percentage of pregnancy IDs in the maternity dataset which were linked with the other three datasets are presented in Fig. 5. The linkage rate for maternity with the neonatal dataset was low because babies only have neonatal records when admitted to neonatal care (e.g. preterm or born in poor condition). The linkage rate of the maternity and activity dataset was affected by one trial site which was unable to provide activity data for any of their women. The linkage rate between the maternity and ultrasound or activity datasets was not expected to be 100% because not all women who give birth in a maternity unit had antenatal care in the same maternity unit (they may have received either no antenatal care, or antenatal care elsewhere).

### Final assessment of data quality

Levels of missing data for key variables required to calculate the trial primary outcome were compared across the linked dataset, following application of the outlier policy, comparing trial arms at two trial phases (pre-randomisation and trial comparison phases). The findings are summarised in Table 5. For neonatal sex and birth weight, there was very little missing data, there was more missing data for maternal BMI, despite infilling missing values in the maternity dataset with data from the ultrasound dataset. For variables with notable levels of missing data, there was variation between trial arms and the period of data collection. Those allocated to the control arm were more likely to have missing BMI data than those in the intervention arm with this difference being more marked in the trial comparison phase as the extent of missingness decreased more in the intervention than control arm over time. By contrast ethnicity was more likely to be missing in the intervention arm than control arm, but the proportion with missing data for both of these variables decreased between the pre-randomisation and trial comparison phases. In the pre-randomisation phase, missing parity data was high (11–15%) in both of the trial arms, this was because we requested parity as an integer (as required to calculate our primary outcome), but it was often only supplied as categorical (nulliparous/multiparous). Nulliparity can easily be converted to parity = 0, but multiparity cannot. These between randomised arm differences in missing data could influence the main trial results and highlight the importance of appropriate methods for dealing with missing data.

**Table 5:** Levels of missing data for key variables after the second data download, comparing trial phases

	Pre-randomisation phase		Trial comparison phase	
	Control	Intervention	Control	Intervention
Maternal height	25.0%	11.0%	20.7%	3.0%
Maternal weight	15.3%	21.6%	12.0%	12.9%
Ethnicity	3.7%	14.1%	3.1%	7.1%
Parity	11.1%	15.2%	15.1%	7.4%
Neonatal sex	0.01%	0.1%	0.1%	0.5%
Neonatal birthweight	0.1%	0.3%	0.3%	0.5%
Gestational age	0.2%	4.8%	1.3%	3.8%

Table 6 shows the distributions for variables that are required for the calculation of the primary outcome, comparing observed data and imputed data by each of the intervention arms. Notably, the proportion of women coded as ‘white’ ethnicity increased during the pre-randomisation phase in the intervention arm; this was expected because one cluster randomised to the intervention, at which the majority of the pregnant women are white, had high levels of missing data on ethnicity during this time period; this was corrected by the time the trial comparison phase data were collected. The proportion of multiparous women increased in the imputed data for the control arm of the trial during both time periods, this was also expected because one control arm cluster only provided information on nulliparity (converted to parity = 0) or multiparity, as a binary value. Parity was therefore only imputed for multiparous women because we knew they had had a baby previously, but not how many. All other summary estimates remained similar.

**Table 6:** Comparison of summary statistics between trial phases and intervention allocation

	Pre-randomisation phase				Trial comparison phase			
	Control sites		Intervention sites		Control sites		Intervention sites	
	Observed data	Imputed dataset	Observed data	Imputed dataset	Observed data	Imputed dataset	Observed data	Imputed dataset
Maternal height (median [IQR])	1.64 [1.60, 1.68]	1.64 [1.60, 1.69]	1.63 [1.58, 1.68]	1.63 [1.58, 1.68]	1.64 [1.60, 1.69]	1.64 [1.60, 1.69]	1.64 [1.60, 1.69]	1.63 [1.59, 1.68]
Maternal weight (median [IQR])	66.0 [58.8, 76.0]	66.0 [58.5, 76.0]	64.5 [57.0, 74.0]	64.7 [57.3, 74.4]	67.2 [59.9, 78.0]	67.0 [59.5, 77.9]	67.2 [59.9, 78.0]	65.4 [58.0, 76.0]
Ethnicity^a^:								
White (%)	62.4%	62.8%	46.9%	50.2%	62.4%	62.7%	50.2%	50.6%
Black (%)	16.6%	16.2%	12.4%	11.8%	15.4%	15.1%	10.8%	10.9%
Asian (%)	13.4%	13.3%	27.1%	24.8%	13.5%	13.5%	25.2%	24.6%
Mixed (%)	2.2%	2.1%	1.6%	1.6%	2.6%	2.6%	1.3%	1.3%
Other (%)	5.4%	5.5%	12.1%	11.6%	6.1%	6.1%	12.5%	12.6%
Parity (% multiparous)	49.4%	53.7%	46.8%	46.5%	48.8%	52.5%	48.8%	51.6%
Neonatal birthweight in grams (median [IQR])	3380 [3050, 3700]	3380 [3050, 3700]	3340 [3010, 3660]	3340 [3010, 3660]	3360 [3040, 3670]	3360 [3035, 3670]	3320 [3000, 3645]	3320 [2996, 3645]
Neonatal gestational age at birth in weeks (median [IQR])	40 [39, 41]	40 [39, 41]	40 [39, 41]	40 [39, 41]	40 [39, 41]	40 [39, 41]	40 [39, 41]	40 [39, 41]

^a^Five categories of ethnicity presented here for brevity, but ethnicity imputed according to categories used by the foetal/neonatal weight customised centile calculator, e.g. British European, Central African, Chinese, Mixed Asian-European

## Discussion

Through this manuscript, we have outlined the data collection and management strategies of the DESiGN Trial, a randomised cluster-control trial which set out to collect quantitative data solely from electronic patient records. We have shown that this method of data collection is feasible and likely to be cost-effective. It would have been much more expensive to collect the same data (e.g. maternal age, weight, height, ethnicity and infant outcomes) on ~ 180,000 pregnancies by employing and training research midwives or research assistants to collect the data in each hospital.

The use of EPR is not without challenges; we have demonstrated that it requires appropriate resources to maximise data completeness and quality. This was improved by having planned an initial data download, followed by a second later download. This allowed the team to test and clarify the first data request and to make improvements that benefited the pre-randomisation data and the data for the two subsequent trial phases. Furthermore, multiple imputation was essential to manage the level of missing data from EPR. It was reassuring to note that summary statistics generated from the imputed dataset were generally concordant with the summary statistics on observed data, differing only where expected because of biases in the missing data. Nevertheless, imputation is based on the untestable missing-at-random assumption and is not guaranteed to reduce bias even if the best possible set of predictors has been chosen. We noted limited instances (reporting of severe perineal trauma, or use of epidural during labour) where missing data were clearly not missing at random, and instead very likely to be negative values. For these, we intend to simply treat missing as negative at analysis as a pragmatic solution.

Another important challenge was in harmonising data received in multiple formats (including free text), to be used for the same purpose. This included heterogeneity of variables names, types and values for the requested data. This arose from the number of different EPR systems that were used for different datasets and was further complicated by some trial sites changing EPR system mid-trial. These issues were less problematic for neonatal data, because Badgernet neonatal is an EPR system which is used universally by neonatal units in the UK, and ultrasound data from the two common EPR systems (Astraia, Germany and Viewpoint, GE Healthcare, USA). This meant that a standardised data extraction query could be made at the start of the trial and was adopted by all sites when downloading data (Badgernet query available in Additional File 1). Our data dictionary (Additional File 2), which provides details of the raw and harmonised variable names, a description of the ideal variable (cleaned) format and instructions on how to re-categorise each categorical variable (from each site) into the harmonised form, could also be extremely helpful to others working in this field.

### Recommendations for the use of EPR in RCTs

We have used our experience to provide the following recommendations to researchers planning trials with a similar EPR data acquisition method. For those considering using EPRs for pregnancy and perinatal research in the UK, we hope that our dictionary will be of value and have provided it in full in the annex.

We suggest discussion with potential trial sites, before recruitment, regarding the required information specification and timelines. This will confirm what data each site is able to provide and makes sure that the researcher and on-site data providers know what is expected of each other.

An on-site local data coordinator (e.g. information technology clinical lead) should be identified, who agrees to take responsibility for liaising with colleagues regarding the generation of reports is likely to expedite the data request process.

We found generation and review of a ‘trial’ data download early in the process very helpful, rather than waiting to download all data at the end of the trial and recommend this process to others. This enabled the trial team to check that all required data was available, obtain a more complete and accurate full data download at the end of the trial and make plans for the data harmonisation process.

We recommend that future groups applying for research funds using EPRs ensure that they are aware of how different sources may record the same data in different ways. Ensure that there is adequate research funding for the on-site data coordinator and other staff involved in providing EPRs as well as adequate funding for the central management of the data, including harmonisation and developing a dictionary.

### Lessons for the future use of EPRs in trials

We used EPRs for DESIGN because we felt it was the only feasible way of conducting a trial including ~ 180,000 pregnancies across 13 maternity units. An alternative approach would have been to use a bespoke trial database in each centre. However, we estimate that this would have required a full-time paid member of research staff at each unit for a minimum of 9 months. Furthermore, staff in the units thought that such an approach would cause a significant burden on them and it could therefore have affected recruitment. Another strategy could have involved data collection using a national reporting system such as the UK’s Hospital Episode Statistics or the UK’s new national Maternity Services Data Set (neither MSDS version was available at the time of planning the trial). Both national datasets have the advantage of data which are already collected to a data specification, which is the same regardless of the site from which data were uploaded. However, the use of these are currently limited by the granularity of information included and the high rates of missing information [16, 17].

As research aims to include more participants so that results are more generalisable across populations and statistical power is increased, we believe the future lies in the greater use of EPRs in clinical trials. For this to happen at scale, national datasets such as those described above, need to be extended using unified data specifications which are sufficiently detailed to collect data required by common core outcome sets and with promotion of data collection quality standards. Established examples of this are the Swedish Medical Birth Register [2], Scottish Medical Record (e.g. SMR02 for maternity records [18]) and the UK National Neonatal Research Database (NNRD) [19]. The UK MSDS is less established than these other examples. As patient records become increasingly switched to EPRs, with inherent clinical coding for diagnosis and procedure codes as well as baseline demographics and characteristics, the quality of EPR data is expected to improve with a resultant quality impact on the ability to report outcomes in clinical trials [20, 21].

## Conclusions

We have demonstrated the feasibility of using EPR to undertake an RCT of 180,000 pregnancies in a situation where data management was complicated by there being no unified EPR system across maternity units. We have shared our experience and provide research recommendations and a detailed data dictionary that will help others in the UK undertaking research with EPRs across maternity units and in non-maternity settings.

## Supplementary Information

**Additional file 1.** Data query for neonatal Badgernet. Code used to generate the dataset from neonatal Badgernet software at each research site.

**Additional file 2.** Data dictionary. Detailed dictionary used for naming variables, describing variables and formatting variable names and values so that they were harmonised across a dataset from 13 sources.

**Additional file 3.** Data variables included in the multiple imputation model. Table of variables explaining the level at which they were imputed and the intended purpose (e.g. characteristics, outcomes).

## Data Availability

The dataset supporting the conclusions of this article is currently not available in a publicly available repository because the data is potentially identifiable.

## Abbreviations

CAG: Confidentiality Advisory Group
DESiGN: Detection of the Small for GestatioNal age fetus
DOB: Date of birth
DOD: Date of delivery
EPR: Electronic patient record
GAP: Growth Assessment Protocol
ID: Identifier
MICE: Multiple imputation with chained equations
MSDS: Maternity Services Data Set
NHS: National Health Service
NNRD: National Neonatal Research Database
SGA: Small for gestational age
SMR: Scottish Morbidity Record
