# Supplementary material for: Using electronic patient records to assess the effect of a complex antenatal intervention in a cluster randomised controlled trial—data management experience from the DESiGN Trial team
Source: Trials. 2021 Mar 8;22:195. doi: 10.1186/s13063-021-05141-8 (PMC7941939; doi:10.1186/s13063-021-05141-8)
Supplement: Supplementary file 1 — Additional file 1. Data query for neonatal Badgernet. Code used to generate the dataset from neonatal Badgernet software at each research site. [file 13063_2021_5141_MOESM1_ESM.docx]

**Badgernet neonatal query for data extraction**

Baby_data query

PARAMETERS [NHSCODE] Text ( 255 );

SELECT NNUEpisodes.BadgerUniqueID AS badger_id, NNUEpisodes.NationalIDMother, NNUEpisodes.HospitalIDMother, NNUEpisodes.NationalIDBaby, NNUEpisodes.HospitalIDBaby, NNUEpisodes.BirthTimeBaby AS Birth_datetime, NNUEpisodes.AdmitTime AS Admission_datetime, NNUEpisodes.Sex AS Neonatal_Sex, NNUEpisodes.GestationWeeks AS GA_weeks, NNUEpisodes.GestationDays AS GA_days, NNUEpisodes.Birthweight AS Birthweight_grams, NNUEpisodes.BirthOrder AS Birth_order, NNUEpisodes.FetusNumber AS Number_of_babies, NNUEpisodes.BirthHeadCircumference AS Head_circumference, NNUEpisodes.Apgar5 AS 5min_apgar, NNUEpisodes.CordArterialpH AS Arterial_cord_pH, NNUEpisodes.CordArterialBE AS Arterial_cord_BE, IIf([Resuscitation] Is Null,Null,IIf([Resuscitation] Is Not Null And [Resuscitation]<>"00","Yes","No")) AS Resp_support_delivery, IIf([DischTime]=Null,"Current inpatient",Int([DischTime]-[AdmitTime])) AS Length_of_stay, [bapm2011 days].[1] AS BAPM2011_IC, [bapm2011 days].[2] AS BAPM2011_HD, [bapm2011 days].[3] AS BAPM2011_SC, IIf([Cooled]=1,"Yes","No") AS Cooling, IIf([HIEGrade]>0,[HIEGrade],Null) AS HIE_worst_grade, diagnoses.Intraventricular_haemorrhage, diagnoses.[Oxygen_required>28days], diagnoses.Necrotising_enterocolitis, diagnoses.Sepsis, diagnoses.Retinopathy_of_prematurity, diagnoses.Hypothermia, diagnoses.Hypoglycaemia, ng_tube_feeding.NG_tube_feeding, NNUEpisodes.DischTime AS Discharge_datetime, Int([dischtime]-[birthtimebaby]) AS Days_at_discharge, IIf([DischargeDestination]="3","Yes","") AS [Neonata_death<28days], NNUEpisodes.DiedCause, [hrg days].[HRG1 IC], [hrg days].[HRG2 HD], [hrg days].[HRG3 SC], [hrg days].[HRG4&5 NC], NNUEpisodes.AdmitFromNHSCode

FROM (((NNUEpisodes LEFT JOIN diagnoses ON NNUEpisodes.EntityID = diagnoses.EntityID) LEFT JOIN ng_tube_feeding ON NNUEpisodes.EntityID = ng_tube_feeding.EntityID) LEFT JOIN [bapm2011 days] ON NNUEpisodes.EntityID = [bapm2011 days].EntityID) LEFT JOIN [hrg days] ON NNUEpisodes.EntityID = [hrg days].EntityID

WHERE (((NNUEpisodes.AdmitFromNHSCode) Like [NHSCODE]));

‘Hrg days’ query

| **Field** | Expr1: NNUDaySum.EntityID | If([HRG]<0,"HRG4&5 NC",Choose([HRG],"HRG1 IC","HRG2 HD","HRG3 SC","HRG4&5 NC","HRG4&5 NC")) | CountOfCareDate: Count(NNUDaySum.CareDate) |
| --- | --- | --- | --- |
| **Table:** |  |  |  |
| **Total:** | Group By | Group By | Expression |
| **Crosstab:** | Row Heading | Column Heading | Value |
| **Sort:** |  |  |  |
| **Criteria:** |  |  |  |
| **Or:** |  |  |  |

Bapm2011 days

| **Field** | Expr1: NNUDaySum.EntityID | f([bapm2011]<0,"Unk",Choose([bapm2011],1,2,3,3)) | CountOfCareDate: Count(NNUDaySum.CareDate) |
| --- | --- | --- | --- |
| **Table:** |  |  |  |
| **Total:** | Group By | Group By | Expression |
| **Crosstab:** | Row Heading | Column Heading | Value |
| **Sort:** |  |  |  |
| **Criteria:** |  |  |  |
| **Or:** |  |  |  |

NG_tube_feeding

| **Field** | Expr1: NNUDaySum.EntityID | ng_tube_feeding: IIf(Sum(IIf([FeedingMethod]="4",1,0))>0,"Yes","") |
| --- | --- | --- |
| **Table:** |  |  |
| **Total:** | Group By | Expression |
| **Sort:** | Ascending |  |
| **Show:** |  |  |
| **Criteria:** |  |  |
| **Or:** |  |  |

Diagnoses

| **Field** | Expr1: NNUCodedItems.EntityID | intraventricular_haemorrhage: IIf(Sum(IIf([Code]="15705" Or [Code]="157 | oxygen_required>28days: IIf(Sum(IIf([Code]="15605",1,0))>0,"Yes","") | necrotising_enterocolitis: IIf(Sum(IIf([Code]="1010683" Or [Code]="10708" Or [Code]="15809",1,0))>0,"Yes","") | sepsis: IIf(Sum(IIf([Code]="1015985" Or [Code]="15007" Or [Code]="15639" Or [Code]="15640" Or [Code]="15641" Or [Code]="15642" Or [Code]="15643" Or [Code]="15644" Or [Code]="15645" Or [Code]="15647" Or [Code]="15648" Or [Code]="15649" Or [Code]="15651" Or [Code]="15653" Or [Code]="15664" Or [Code]="15671" Or [Code]="15679" Or [Code]="16776",1,0))>0,"Yes","") | retinopathy_of_prematurity: IIf(Sum(IIf([Code]="11010232" Or [Code]="15218" Or [Code]="15219" Or [Code]="15220" Or [Code]="15221" Or [Code]="15222" Or [Code]="15223" Or [Code]="15224",1,0))>0,"Yes","") | hypothermia: IIf(Sum(IIf([Code]="15826",1,0))>0,"Yes","") | hypoglycaemia: IIf(Sum(IIf([Code]="15773",1,0))>0,"Yes","") |
| --- | --- | --- | --- | --- | --- | --- | --- | --- |
| **Table:** |  |  |  |  |  |  |  |  |
| **Total:** | Group By | Expression | Expression | Expression | Expression | Expression | Expression | Expression |
| **Sort:** |  |  |  |  |  |  |  |  |
| **Show:** |  |  |  |  |  |  |  |  |
| **Criteria:** |  |  |  |  |  |  |  |  |
| **Or:** |  |  |  |  |  |  |  |  |
