# Supplementary material for: Using electronic patient records to assess the effect of a complex antenatal intervention in a cluster randomised controlled trial—data management experience from the DESiGN Trial team
Source: Trials. 2021 Mar 8;22:195. doi: 10.1186/s13063-021-05141-8 (PMC7941939; doi:10.1186/s13063-021-05141-8)
Supplement: Supplementary file 3 — Additional file 3. Data variables included in the multiple imputation model. Table of variables explaining the level at which they were imputed and the intended purpose (e.g. characteristics, outcomes). [file 13063_2021_5141_MOESM3_ESM.docx]

## Additional File 3

### Data variables included in the multiple imputation model

| Variable | Level of imputation | Predictors |
| --- | --- | --- |
| Index of Multiple Deprivation (IMD) | Within cluster | Age*, ethnicity, parity, gestational age at birth, birthweight, mode of birth, estimated blood loss, number of ultrasound scans >24 weeks’, SGA detected, time period |
| Maternal age at estimated conception | Within cluster | IMD, ethnicity, parity, gestational age at birth, birthweight, mode of birth, estimated blood loss, number of ultrasound scans >24 weeks’, SGA detected, time period |
| Maternal ethnicity (customised groups) | Within cluster | IMD, age*, parity, gestational age at birth, birthweight, mode of birth, estimated blood loss, number of ultrasound scans >24 weeks’, SGA detected, time period |
| Maternal Height | Across clusters | IMD, age*, ethnicity, parity, gestational age at birth, birthweight, mode of birth, estimated blood loss, number of ultrasound scans >24 weeks’, SGA detected, time period |
| Maternal Weight | Across clusters | IMD, age*, ethnicity, parity, gestational age at birth, birthweight, mode of birth, estimated blood loss, number of ultrasound scans >24 weeks’, SGA detected, time period |
| Parity (ordinal) | Across clusters | IMD, age*, ethnicity, gestational age at birth, birthweight, mode of birth, estimated blood loss, number of ultrasound scans >24 weeks’, SGA detected, time period |
| Gestational age at birth | Within cluster | IMD, age*, ethnicity, parity, birthweight, mode of birth, estimated blood loss, number of ultrasound scans >24 weeks’, SGA detected, time period |
| Birthweight | Within cluster | IMD, age*, ethnicity, parity, gestational age at birth, mode of birth, estimated blood loss, number of ultrasound scans >24 weeks’, SGA detected, time period |
| Onset of labour | Within cluster | IMD, age*, ethnicity, parity, gestational age at birth, birthweight, mode of birth, estimated blood loss, number of ultrasound scans >24 weeks’, SGA detected, time period |
| Mode of birth | Within cluster | IMD, age*, ethnicity, parity, gestational age at birth, birthweight, estimated blood loss, number of ultrasound scans >24 weeks’, SGA detected, time period |
| Estimated blood loss | Within cluster | IMD, age*, ethnicity, parity, gestational age at birth, birthweight, mode of birth, estimated blood loss, number of ultrasound scans >24 weeks’, SGA detected, time period |
| Number of antenatal appointments after 24 weeks’ gestation | Within cluster | IMD, age*, ethnicity, parity, gestational age at birth, birthweight, mode of birth, estimated blood loss, number of ultrasound scans >24 weeks’, SGA detected, time period |
| Number of ultrasound scans after 24 weeks’ gestation | Within cluster | IMD, age*, ethnicity, parity, gestational age at birth, birthweight, mode of birth, estimated blood loss, number of ultrasound scans >24 weeks’, SGA detected, time period |
| Total length of antenatal stay after 24 weeks’ gestation (per pregnancy) | Within cluster | IMD, age,* ethnicity, parity, gestational age at birth, birthweight, mode of birth, estimated blood loss, number of ultrasound scans >24 weeks’, SGA detected, time period |
| Total length of postnatal stay (per pregnancy) | Within cluster | IMD, age*, ethnicity, parity, gestational age at birth, birthweight, mode of birth, estimated blood loss, number of ultrasound scans >24 weeks’, SGA detected, time period |
| SGA detected (SGA detected, SGA not detected, no SGA) | Within cluster | IMD, age*, ethnicity, parity, gestational age at birth, birthweight, mode of birth, estimated blood loss, number of ultrasound scans >24 weeks’, SGA detected, time period |
| *Maternal age at estimated conception (38 weeks prior to the estimated due date of the baby) | | |
